# Supplementary material for: Impact of the suboptimal communication network environment on telerobotic surgery performance and surgeon fatigue
Source: PLoS One. 2022 Jun 16;17(6):e0270039. doi: 10.1371/journal.pone.0270039 (PMC9202925; doi:10.1371/journal.pone.0270039)
Supplement: S1 Table — (DOCX) [file pone.0270039.s001.docx]

| **Depth perception** | | | | |
| --- | --- | --- | --- | --- |
| １ | 2 | 3 | 4 | 5 |
| Constantly overshoots target, wide swings, slow to correct |  | Some overshooting or missing of target, but quick to correct |  | Accurately directs  instruments in the correct plane to target |
| **Bimanual dexterity** | | | | |
| 1 | 2 | 3 | 4 | 5 |
| Uses only one hand, ignores nondominant hand, poor coordinate |  | Uses both hands, but does not optimize interaction between organized |  | Expertly uses both hands in a complementary way to provide best exposure |
| **Efficiency** | | | | |
| 1 | 2 | 3 | 4 | 5 |
| Inefficient efforts; many uncertain movements; constantly changing focus or persisting without progress |  | Slow, but planned movements are reasonably organized |  | Confident, efficient and safe conduct, maintains focus on task, fluid progression |
| **Force sensitivity** | | | | |
| 1 | 2 | 3 | 4 | 5 |
| Rough moves, tears tissue, injures nearby structures, poor control, frequent suture breakage |  | Handles tissue reasonably well, minor trauma to adjacent tissue, rare suture breakage |  | Applies appropriate tension, negligible injury to adjacent structures, no suture breakage |
| **Autonomy** | | | | |
| 1 | 2 | 3 | 4 | 5 |
| Unable to complete entire task, even with verbal guidance |  | Able to complete task safely with moderate guidance |  | Able to complete task independently without prompting |
| **Robotic control** | | | | |
| 1 | 2 | 3 | 4 | 5 |
| Consistently does not optimize view, hand position, or repeated collisions even with guidance |  | View is sometimes not optimal. Occasionally needs to relocate arms. Occasional collisions and obstruction of assistant |  | Controls camera and hand position optimally and independently. Minimal collisions or obstruction of assistant |

**S1 Table. Global Evaluative Assessment of Robotic Skills (GEARS)**

Source: Goh AC, Goldfarb DW, Sander JC, Miles BJ, Dunkin BJ (2012) Global Evaluative Assessment of Robotic Skills: Validation of a Clinical Assessment Tool to Measure Robotic Surgical Skills. J Urol 187:248
