# Supplementary material for: Impact of the suboptimal communication network environment on telerobotic surgery performance and surgeon fatigue
Source: PLoS One. 2022 Jun 16;17(6):e0270039. doi: 10.1371/journal.pone.0270039 (PMC9202925; doi:10.1371/journal.pone.0270039)
Supplement: S2 Table — (DOCX) [file pone.0270039.s002.docx]

**S2 Table: Piper Fatigue Scale-12（PFS-12）**

| **Piper Fatigue Scale (PFS-12)** |
| --- |
| Directions: Please circle the number which best describe the fatigue you are experiencing  **IN THE PAST 4 WEEKS.**   1. To what degree is the fatigue are you feeling with your ability to complete your work activations?   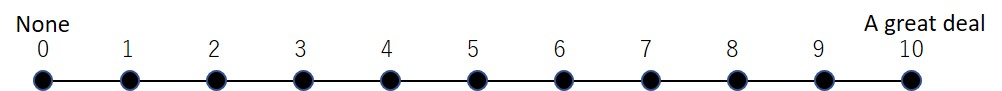   1. Overall, how much is the fatigue which you are experiencing interfering with your ability to engage in the kind of activities you enjoy?   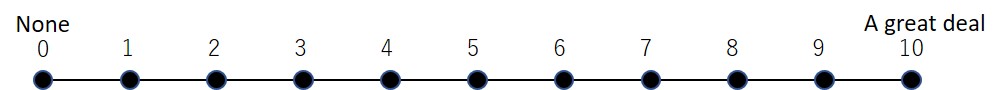   1. How would you describe the degree of intensity or severity of the fatigue which you are experiencing?   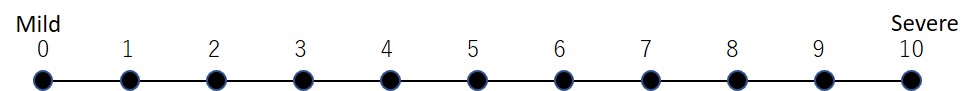   1. To what degree would you describe the fatigue which you are experiencing as being?   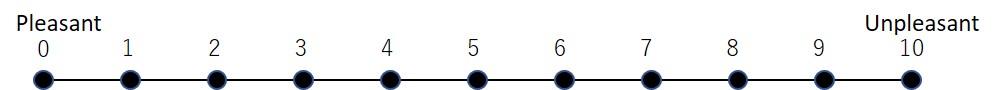   1. To what degree would you describe the fatigue which you are experiencing as being?   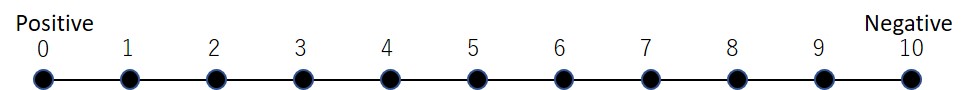   1. To what degree would you describe the fatigue which you are experiencing as being?   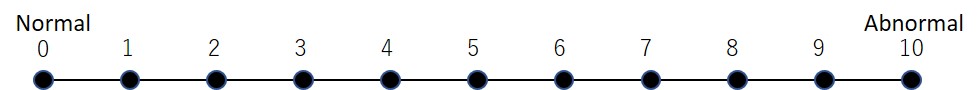   1. To what degree are you feeling?   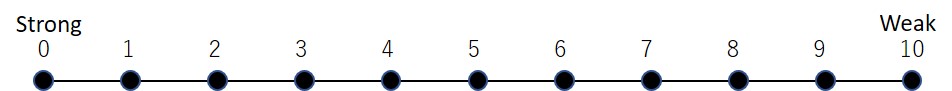   1. To what degree are you feeling?   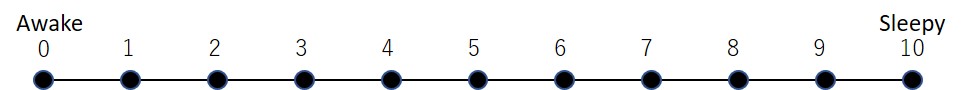   1. To what degree are you feeling?   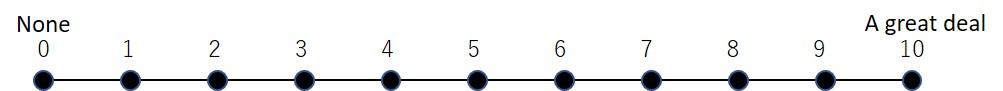   1. To what degree are you feeling?   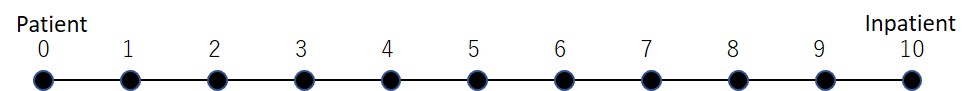   1. To what degree are you feeling?   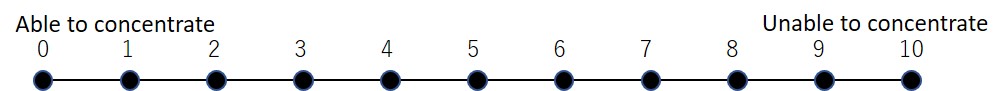   1. To what degree are you feeling?   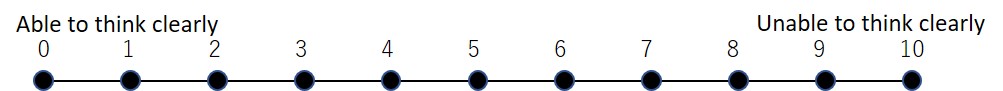 |

Piper Fatigue Svale-12 (PFS-12) Scoring Directions:

1) All scores should be reported as the higher the number/ score, the worse the fatigue.

2) There are no scoring reversals.

3) There are four subscale scores with 3 items each and a Total Fatigue Score

a. Behavioral

- #1 [Interference with work/school activities]
- #2 [Interference with enjoyable activities] – note new wording
- #3 [Fatigue intensity/severity]

b. Affective

- #4 [Pleasant/unpleasant]
- #5 [Positive/negative]
- #6 [Normal/ abnormal]

c. Sensory

- #7 [Strong/weak]
- #8 [Awake/sleepy]
- #9 [Refreshed/tired]

d. Cognitive

- #10 [Patient/impatient]
- #11 [Ability to concentrate]
- #12 [Ability to think clearly]

5) Subscale items are summed and divided 3 to keep the score on a 0-10 scaling. If 2 out
of 3 subscale items have been answered, mean item subscale substitution can be used to fill in the missing item

6) To calculate the PFS-12 Total Score: sum all 12 items and divede by 12.

7) In addition, cut-points/ categories have been empirically derived for the PFS-12 total
fatigue score:

- None = 0
- Mild = 1-3
- Moderate = 4-6
- Severe = 7-10

Source: Reeve BB, Stover AM, Alfano CM, Smith AW, Ballard-Barbash R, Bernstein L, McTiernan A, Baumgartner KB, Piper BF (2012) The Piper Fatigue Scale-12 (PFS-12): Psychometric findings and item reduction in a cohort of breast cancer survivors. Breast Cancer Res. Treat. 136: Appendix
