# Supplementary material for: Impact of the suboptimal communication network environment on telerobotic surgery performance and surgeon fatigue
Source: PLoS One. 2022 Jun 16;17(6):e0270039. doi: 10.1371/journal.pone.0270039 (PMC9202925; doi:10.1371/journal.pone.0270039)
Supplement: Attachment — Submitted filename: Response to Reviewers.docx [file pone.0270039.s004.docx]

Responses to Reviewers

Responses to the Comments by the Associate Editor:

*1. Please review your reference list to ensure that it is complete and correct. If you have cited papers that have been retracted, please include the rationale for doing so in the manuscript text, or remove these references and replace them with relevant current references. Any changes to the reference list should be mentioned in the rebuttal letter that accompanies your revised manuscript. If you need to cite a retracted article, indicate the article’s retracted status in the References list and also include a citation and full reference for the retraction notice.*

Reply:
Thank you very much for your helpful comment. We confirmed that the reference list is complete and correct. Also, there was no retracted reference.


Responses to the Comments by Reviewer 1:

*1) Thank you for your response to my comments.
In response to the second comment, "We have added text to the Results section to clarify this.(Page 16-17, Lines 267-269)", but unfortunately I could not find the changed section.*

Reply:
Thank you for pointing this out. We are very sorry that there was an incorrect statement in the description of the modification. We revised the description regarding overshooting as follows.
Overshooting due to communication delay was not observed. We evaluated it using GEARS, which has an evaluation item that includes the concept of overshooting of forceps called “Depth perception.” There was no significant difference in “Depth perception” by line (3.7 vs. 3.5, p = 0.35). Furthermore, we have added text to the Results section to clarify this (Page 17, Lines 270-272).
